# “If he's in pain i'm also in pain”: a qualitative study on support needs of significant others of persons with rheumatoid arthritis

**DOI:** 10.1080/17482631.2026.2732696

**Published:** 2026-09-16

**Authors:** Maria Bergström, Åsa Larsson Ranada, Annette Sverker, Mathilda Björk

**Affiliations:** a Department of Health, Medicine and Caring Sciences, Linköping University, Linköping, Sweden; b Clinical Department of Pain and Rehabilitation Medicine in Linköping, Region Östergötland, Linköping, Sweden

**Keywords:** Delivery of healthcare, rehabilitation, rheumatoid arthritis, significant others, social support

## Abstract

**Purpose:**

As rheumatoid arthritis (RA) affects both the patient and their significant others, the aim of this study is to describe the views and experiences of significant others of persons with contemporary treated RA.

**Methods:**

Patients at rehabilitation clinics in southeast Sweden were asked to invite significant others to participate in individual semi-structured interviews. Sixteen significant others accepted. Data were analyzed using Braun and Clarke's reflexive thematic analysis.

**Results:**

Experiences were described through three themes: *1) A need to be involved and informed:* significant others described how they were provided information, but also felt left out and uninformed; *2) Help and support go both ways:* patients and their significant others were big supports to each other; however, significant others were in need of more support themselves; *3) Making everyday life work:* they had found routines in everyday life, but had lost certain activities and, experienced pressure to adopt a caring role.

**Conclusions:**

Significant others of persons with RA report lacking information and limited involvement in rehabilitation. In order for significant others to provide the best possible support, attention should be paid to their needs. If given the right conditions, significant others can potentially reduce the burden on the healthcare system.

## Introduction

Rheumatoid arthritis (RA) is a chronic inflammatory disease, affecting approximately 5 per 1000 people worldwide, with symptoms such as pain, fatigue, and swollen joints (Aletaha & Smolen, 2018). Additionally, significant others of the individuals with the diagnosis are affected in their everyday life (Untas et al., 2020). However, national guidelines regarding RA (National Board of Health & Welfare, 2021) fail to explicitly involve significant others in treatment, follow-ups, and rehabilitation, which goes against, for example, the Swedish Patient Act (Ministry of Health & Social Affairs, 2014).

Research concerning significant others often focuses on the patient, with significant others only included as a secondary focus. Although research has recently been conducted regarding partners of people with inflammatory arthritis. This Danish study (Hansen et al., 2026a) showed that partners experience emotional burden, such as feelings of powerlessness and sorrow, worries about the future, and impact on intimate life. Furthermore, being the partner of a person with inflammatory arthritis was described as a learning process. However, significant others in this case are restricted to partners exclusively. Persons with RA have described significant others as facilitators, but also as having a lack of understanding (Kostova et al., 2014). For example, significant others can both over- and underestimate the patient's pain, which in both cases can constitute a hindrance (Lehman et al., 2011). Significant others themselves express a lack of knowledge (Tiwana et al., 2015), but also a lack of support from healthcare organizations (Fallatah & Edge, 2015). When evaluating the implementation of national guidelines, differences in preconditions for team rehabilitation were displayed. These differences included lack of follow-up routines or written follow-ups instead of oral instructions (National Board of Health & Welfare, 2024), which might result in missed updates and missed discussions between healthcare providers and patient, and conclusively, also significant others. According to Untas et al. (2020), limited insights might lead to disruptions in everyday life, since persons with RA can expect understanding and knowledge from their significant others, yet significant others lack access to this knowledge. In addition, significant others themselves express their role as an occasional mental burden (Matheson et al., 2010). Furthermore, both significant others and persons with RA express difficulties in everyday life, such as making plans and, worrying about the future (Brignon et al., 2020).

A recent review aiming to explore significant others' potential in relation to the management of inflammatory arthritis, further concluded that a greater focus on significant others might improve self-management skills, as well as address the needs of the significant others (Hansen et al., 2024). However, included publications often target the patient and the significant other together as a unit, limiting the possibility of highlighting the significant other's own unique point of view.

Back in 2010, Matheson and colleagues described a restricted life as well as bigger necessary adaptations in individuals' lives and their role as significant others for a person with RA (Matheson et al., 2010). Since then, the guidelines for treatment of RA have been updated (Smolen et al., 2026), and persons with RA are better equipped to, for instance, continue working life. For example, biological treatment has led to better outcomes regarding disease activity and function impairments (Shadick et al., 2019). In this sense, the conditions for the patients have changed and, consequently, the role of the significant others may have shifted, becoming less focused on practical help and caregiving (Bergström et al., 2023). However, everyday life is affected even after many years with RA (Bergström et al., 2025), and this in turn also affects the significant others of persons with RA through, for example, changed routines and limited activities.

In all, this calls for an updated outlook on the view of significant others of persons with RA. The aim of this study is therefore to describe the views and experiences of significant others of persons with contemporary treated RA.

## Materials and methods

### Study design

This study undertook a qualitative design with individual semi-structured interviews with significant others of persons with RA, where the material was analyzed through thematic analysis as described by Braun and Clarke (2006, 2019, 2023). Through the analysis process, researcher subjectivity was acknowledged and viewed as an inevitable resource (Braun & Clarke, 2023). The Standards for Reporting Qualitative Research (SRQR) reporting checklist (O’Brien et al., 2025) was used when developing the manuscript.

Following the updated European Alliance of Associations for Rheumatology (EULAR) recommendation for the involvement of patient research partners (de Wit et al., 2024), two research partners have been involved in the project to ensure the relevance of the results. They have been involved throughout the process, starting with the research question, and following all the way through the discussion and final version of the manuscript. Particularly, they were involved in discussions regarding the results and clinical implications. Both research partners have personal experiences of being a significant other and, have gone through an appropriate research partner training course. As our research partners are not patients, they have provided their knowledge and experiences from their area of expertise—in this case, the significant others' point of view.

### Researcher positioning and reflexivity

The research team comprises four women with professional backgrounds in occupational therapy and social work. The team has extensive experience of conducting qualitative research in areas such as clinical research and health care, including people with rheumatic diseases. The researchers have conducted several studies within this field and have therefore developed substantial prior understanding of the research context. The researchers' joint epistemological view acknowledge the subjective experiences of the participants as central throughout the research process. Furthermore, the different backgrounds and experiences are inevitably brought into the analysis. For example, working with people with RA influence researchers' individual outlook on themes. However, different approaches to data are considered a strength (Braun & Clarke, 2019).

### Participants

In southeast Sweden, a longitudinal cohort named TIRA-2 (Early Interventions in Rheumatoid Arthritis) (Thyberg et al., 2005) included persons with RA between 2006 and 2009. Five rheumatology clinics were involved in the cohort and invited patients at the time of their diagnosis. Approximately ten years after diagnosis, patients from this cohort were approached and asked to invite a significant other (as defined by the patient, se further explanation below) to an individual interview between 2018 and 2019. This means that these patients have lived with the diagnosis for more than a decade, indicating that the diagnosis was established and a routine part of their everyday life.

In this study, a significant other refers to “a person who is important to one's well-being” (Merriam-Webster, 2026). The choice of this definition originates from a person-centred view where the patient him- or herself defines who is considered to be a significant other. In other words, a significant other in this study does not automatically encompass a close family member. Originally, 31 persons with RA were approached to invite a significant other, as defined by the patient. Sixteen significant others agreed to participate in this interview study and gave their written consent. This number of participants was considered reasonable from the aspect of information power (Malterud et al., 2016), a concept that aligns with thematic analysis (Braun & Clarke, 2021). Most of the participants were female (*N* = 11), and most were partners (*N* = 12) of persons with RA. However, the participants also consisted of a parent, two children, and a close friend. Most of the participants had long-lasting relationships with the persons with RA, up to 54 years (median 41 years). Participants are presented in Table I.

**Table I.** Participant characteristics.

Code	Sex	Age (years)	Relationship	Length of relationship (years)
1	Male	52	Partner	10
2	Female	47	Partner	29
3	Female	27	Partner	7
4	Female	48	Daughter	48
5	Female	74	Wife	14
6	Female	39	Daughter	39
7	Male	61	Partner	21
8	Female	62	Wife	46
9	Female	71	Mother	44
10	Female	60	Wife	41
11	Female	67	Close friend	52
12	Male	69	Husband	44
13	Male	77	Husband	54
14	Female	57	Wife	41
15	Female	69	Wife	48
16	Male	69	Partner	29

### Data collection

An interview guide (available upon request) was constructed with focus on the experiences of being a significant other to a person with RA. The interview guide was developed using a semi-structured format with key topic areas introduced by opening questions and explored through follow-up questions. Questions from the interview guide concerned topics such as expectations on one's role as a significant other. Additionally, the initial question “How do you experience being a significant other of a person with RA?” started the interviews. The interview guide was tested in a pilot interview prior to data collection. After the pilot interview, the order of the questions was slightly adjusted. The data from the pilot interview are not part of this study. The participants chose the time and place of the interview. All interviews were conducted in a private setting that was familiar to the participant, in most cases the participant's home. The interviews took place between November 2018 and November 2019, and lasted between 17 and 50 min (median 42 min). All interviews were conducted face-to-face, were audio-recorded using a digital voice recorder, and transcribed verbatim and anonymized. Two researchers (both registered occupational therapists experienced in qualitative research) conducted the interviews. None of the researchers had previous connections with the participants and were not involved in the rehabilitation of the patients.

### Data analysis

To shed light on the experience of being a significant other of a person with RA, reflexive thematic analysis with a semantic approach (i.e., inductively conducted based on what the participants said in the interviews) according to Braun and Clarke (2006, 2019, 2023) was chosen. Thus, as the participants' subjective experiences are at the forefront, a constructive research paradigm has been applied. The analytical process was guided by dialogic reflexivity (Braun & Clarke, 2023), whereby interpretations were critically examined and refined through discussions of differing perspectives and interpretations during research team meetings. In accordance with the first phase—familiarization—two authors (MBe, ÅLR) read through the interviews and took notes. Discussions about findings lead to the second phase where initial codes were generated. Afterwards, the material was read through again with the initial codes in mind.

Data extracts were gathered by MBe and ÅLR, and condensed and coded by MBe (who was not involved in the data collection). During this step, new codes were added. MBe and ÅLR then checked all codes together to ensure that both perspectives were considered in the interpretation of the codes. Then, in the third phase, codes were grouped into different initial themes and checked again. To facilitate this phase, a thematic map was used. Next, MBe and ÅLR discussed potential themes before the fourth phase followed. In this phase, co-authors and two research partners read two interviews each, as well as the suggested themes, which was followed by a discussion involving all authors and the two research partners. This approach was chosen with the co-authors' and research partners' different backgrounds in mind. The major role of the two research partners was to ensure the relevance of the results by sharing their experiences as significant others. Additionally, they contributed to both enriching and challenging the interpretation of themes during analysis. As both co-authors and research partners shared the view of the overall results, no further themes were added. However, minor adjustments were made as some aspects of the results were visible across more than one theme. In the fifth phase, MBe suggested names and short definitions of the themes, which were discussed with co-authors and research partners. During the analysis process, a reflexive approach was employed (Braun & Clarke, 2006, 2019, 2023) and the process was featured by discussions between authors, particularly MBe and ÅLR.

### Ethical considerations

All participants were informed about the aim of the study and their right to withdraw without specific reasons. They all gave written consent to take part in this study. The study was conducted in accordance with the Declaration of Helsinki (World Medical Association, 2025) and has been approved by the Regional Ethics Committee at Linköping University, Dnr 2018/158-31, 2019-00733.

## Results

The experiences of being a significant other of a person with RA is described through three themes: 1) A need to be involved and informed, 2) Help and support go both ways, and 3) Making everyday life work. These themes illuminate the views of significant others.

### A need to be involved and informed

A major part of the material presented views on whether significant others of persons with RA experience enough information and involvement in the diagnosis and treatment. This was a two-sided coin where several of them did not feel that they were involved enough; e.g., they did not attend clinical visits and were not included in conversations with healthcare professionals. On the other hand, significant others also felt more informed and involved, for example, when the persons with RA shared information.

In cases where significant others did not feel involved, this could be due to the fact that persons with RA tended not to share new information from follow-ups. Furthermore, significant others could have limited knowledge about the diagnosis, which added to the uncertainty.

*“The only thing I really know is that she’s in pain. Ache sometimes. That she gets so-called flares, that it kind of gets worse for a while before it gets better. I don’t know much more than that”.* Code 1

One strategy used by the significant others, to keep as updated as possible regarding the patient's situation, was to read between the lines. Since most of the relationships were decades-long, they knew each other well. The significant others were able to understand when the persons with RA were in pain or unwell, often based on the mood.

*“You learn as you go…I mean I can see when he is in pain.”* Code 10

An uncertainty regarding when to push and when to stop was discussed. In other words, when to encourage or “let” the person with RA handle heavy tasks, and when to take a step back or take over tasks themselves. This was connected to whether there was a preunderstanding of the diagnosis or not. Limited knowledge hindered appropriate action. Also, even though relief eventually followed, significant others described an initial worry directly after diagnosis. This was often due to misconceptions about the diagnosis itself. For example, more severe impairments were expected, and treatment with chemotherapy was associated with other types of diagnoses, which could increase their concern. Furthermore, information from the healthcare system was lacking.

*“Yes, that [information meeting] scared her and also me because I saw what she was like when she came back. So, it was very unsettling after that for a while, but now for the past few years she´s been doing great.”* Code 9

Furthermore, these types of misconceptions hindered the significant others' possibility to take an active role. With more and better information, more support could have been provided. For example, a daughter described how she had not been involved in visits to physiotherapy or occupational therapy, and therefore had limited opportunities to support her mother:

*”I have not been part of it and not been given any information either. So, I cannot push her in whatever exercises she is supposed to do.”* Code 4

When significant others described involvement, some also stated that they felt more involved at the start of the diagnosis, but later on they were a bit forgotten. This also includes significant others wishing for more information and the opportunity to attend clinical visits. One partner described how she went through lists of new medication when possible, and some described that they had been present, but not during the actual visit.

*“I have been present once and then I sat in the waiting room.”* Code 8

As significant others call for more information; it is, for example, suggested to be in the form of a three-part conversation, with both the person with RA and the significant other present during the clinical visits. Additionally, there is a wish to be more explicitly invited to clinical visits; for example, being “told” to participate, rather than just “invited”.

*”Would not be a bad idea to have someone next to you maybe to a doctor’s appointment. One of these longer appointments, or that you at least were invited to join”* Code 2

### Help and support go both ways

Helping and supporting each other was described as natural and obvious to most participants. They expressed feeling supported by the persons with RA. It was often clear that the person with RA also cared for the significant other and that he/she was not necessarily the one exclusively in need of support.

*“No but that you push each other and that you are there when someone needs to talk, that you are present, you are there for one another, I think so.”* Code 3

This concept of support going both ways was exemplified through dividing chores at home, being a good listener, taking turns when driving a car, carrying heavier objects, helping out with pets, and being an affectionate partner.

However, a feeling of one-sided support was also expressed, in that the significant other tries to “lift” the person with RA. This could take the form of both physical assistance as well as mental support. In these cases, some frustration and sadness surfaced, as exemplified by a daughter to a woman with RA:

*“And sometimes I can feel that it gets tough to always need to be the one who lifts her up and handle this.”* Code 4

Some significant others also expressed a feeling of “not being enough”, meaning that they would want to and need to be a bigger support to the persons with RA, but that they had their own limitations. Examples of this could be not taking on many domestic responsibilities, or a feeling of not having enough patience.

Furthermore, significant others themselves could need support during different phases of the diagnosis. For example, shortly after diagnosis or during the first period with RA, as this could be a confusing and uncertain time with a lot to process. Yet, this support was not offered by the healthcare system. Instead, it was found among friends or family members.

*“Not from the healthcare system, no. But it was more that you talked to your friends and things like that, and…yes. Sort of to ease the burden because you were maybe a bit sad at first.”* Code 5

However, a continuous need of support in different forms was described after many years. For example, a daughter of a woman with RA articulated an increased need to “nag” and someone who listened to her unloading. Moreover, issues were raised regarding the best possible individual to turn to in such cases. For example, a significant other might turn to a close family member with concerns, but the family member might not have the same view or information as the significant other. In the interviews, it is suggested that others in the same situation might be of greater importance when it comes to support. This can have a positive impact on the well-being of significant others.

*“Well, to hear other relatives how they are doing. No but I could imagine that actually, to participate of there ever existed any [groups].”* Code 10

The concept of help and support can also be seen in a different light, as the significant others described their intentions of saving the patient's energy. This could be done by encouraging the patient to slow down, avoid certain activities and, sometimes, ask for help instead. Although, for this to be an actual help and support, significant others would need to have knowledge about the diagnosis and the ability to interpret the situation correctly. Otherwise, it might lead to unnecessary precautions if the person with RA is asked to step away from activities that are not harmful, which in turn might lead to the person with RA feeling down. This is well demonstrated in the following quote:

*“Support to me, then I think that I should not take over. I shall support her with what I know she cannot manage, but what she can manage she will handle on her own, to maintain what she actually can.”* Code 4

### Making everyday life work

In the big picture, everyday life was either perceived as affected to some amount or not affected at all. This could also vary during the period of the diagnosis. One type of impact was that the significant other was worried about the patient and that it could be difficult to see a person who was dear to them and important in their lives feeling ill.

*“Yes I feel good as long as [husband] feels good. If he’s in pain I’m also in pain…”* Code 14

Significant others might also experience certain demands in their role, both from the patient but also from others, such as family or friends. For example, that the significant other is expected to take on a particular caring role. These types of demands were rarely sensed from the persons with RA, but rather from friends.

*“Well…sometimes you might feel that our friends are expecting that too, of course you do.”* Code 12

The fact that the persons with RA could no longer participate in all previous activities also impacted everyday life, and was something they had to adapt to. One participant gave a specific example of giving up the common interest of hiking due to the diagnosis, but limitations in general were also mentioned.

Depending on the type of relationships, different demands were identified. For example, if they were a couple living together, or a parent-child relationship who did not share a household, the situation was perceived differently. In this study, the significant others who were partners of persons with RA expressed more frustration and a feeling of helplessness. Some differences between men and women in the significant other's role were also observed. Men more seldom expressed worries about the person with RA's well-being, compared to women, and were interpreted as more observers than an active partner.

In the following quote, a male partner expressed impact on everyday life, and that adaptations had not been necessary:

*“To be perfectly honest, I don’t really notice a big difference, you know. She kind of is like that, even if she’s in pain…”* Code 1

This can be compared to a statement from a wife, who expressed her worry regarding her husband's condition:

*“No, but sometimes I can feel worried that it will get much worse.”* Code 15

Another aspect is the question of generational differences. For example, partners who were similar in age seemed more willing to accept help from one another, compared to a mother who did not want to interfere in her daughter's life too much. This added a further layer to the matter of impact on everyday life, and making everyday life work. When the persons with RA accepted assistance, everyday life could function more seamlessly.

As previously mentioned, significant others disclosed some expectations or demands placed on them in their role as a significant other. However, many participants expressed that they did not feel any expectations placed on them by the person with RA or others.

*“No, no more demands than what should be expected in a daughter-mother relationship.”* Code 6

In this case, the RA diagnosis had not interrupted their relationship. Furthermore, participants expressed a feeling of being able to continue with their lives and that activities were being adapted without further difficulties. For example, as described by the partner of a man with RA:

*“We still have…kind of continued our lives, pretty much like we did before, so, he can still walk the dog, once or twice a day, take those long walks…”* Code 2

These are examples of a minimal impact in everyday life of the participants, both in regard to relationships and roles, as well as the number of adaptations needed in their everyday life. They had simply made their life with the diagnosis work.

## Discussion

Being a significant other of a person with RA is experienced as impacting everyday life in several ways which are described in the presented themes as a need to be involved and informed, that help and support go both ways, and finding a way to make everyday life work.

The question of involvement and information touches the subject of patient education. As stated by Jones et al. (2022), the recommendation regarding inflammatory arthritis is that patient education should systematically and repeatedly be proposed throughout the disease course, and preferably delivered in groups. This type of education modules is transferable to significant others. The provision of repeated information and the possibility of discussing the situation with peers are very much in line with the wishes of this study's participants. Furthermore, patients and significant others participating in such interventions as a unit has demonstrated positive outcomes. For example, family members of persons with inflammatory rheumatic diseases are suggested to participate in interventions, as they reportedly affect the patients' adaptations to the illness (Dimitraki et al., 2023). Interventions where patients and a partner/spouse/sibling/close friend, etc., participate together have also been reported to result in significantly better pain and disability outcomes; however, only in the short-term and based on evidence of moderate quality (Fritsch et al., 2021). Nevertheless, significant others being involved in rheumatology care and rehabilitation have previously been highlighted. Pile et al. (2020) report that active participation in consultations and treatment decisions was considered important by both persons with RA and their carers. Yet, both patients, carers and rheumatologists believed that the carers did not receive enough support. This support and information about diagnosis and treatment would, in most cases, preferably to be provided by the healthcare system. Furthermore, a recent qualitative study describes how partners of persons with inflammatory arthritis gain a better understanding from direct information at clinical visits than from other sources, such as online searches (Hansen et al., 2026b). Our study's results further emphasize the importance of involving and informing significant others in treatment and rehabilitation and acknowledge their own needs for support. As involvement of significant others is common in other types of diagnoses, such as people with dementia (Hayward et al., 2022), and persons with mental illness (Ewertzon & Hanson, 2019), we see no reason why this should not be applicable and more thoroughly implemented in RA practice.

Support as such is extensively proven important. For example, people with inflammatory arthritis describe how support from family and friends have a positive impact on their mental well-being (Abild et al., 2024). Furthermore, in persons with musculoskeletal diagnoses, statistically significant positive correlations have been found between pain self-efficacy and support from significant others (Anyfantopoulou & Theofilou, 2023). On the other hand, excessive support from partners to persons with RA can instead lead to a sense of helplessness (Čepukienė & Puzerienė, 2024) once again highlighting the importance of significant others receiving information in order to provide the right type of support to the person with RA. Furthermore, if significant others are given the opportunity to be well-informed and prepared, their positive impact has the possibility of reducing the need for considerable healthcare interventions. Another aspect of support is the transition from physical assistance to more focus on emotional support (Bergström et al., 2023), following effective treatment. Emotional support has been identified as particularly important in inflammatory arthritis (Brignon et al., 2020) and emotional support from spouses has been associated with decreased pain in persons with RA (Pow et al., 2018). Therefore, greater emphasis on emotional support is warranted when involving significant others more thoroughly in RA rehabilitation.

That help and support go both ways shows the complexity of relationships where one partner has an illness. This can be viewed in terms of dyadic coping, the process of coping that transpires between couples challenged by one partner's illness. Mittinty et al. (2024) report that both persons with RA and their spouses affect each other's well-being when engaging in dyadic coping. Furthermore, spouses perceiving more supportive dyadic coping also reported better relationship quality. Likewise, persons with RA receiving more supportive dyadic coping also reported lower levels of depression, anxiety, and stress, as well as higher relationship quality. In other words, dyadic coping benefits the well-being of both persons with RA and significant others. Although acknowledging new roles—and the loss of roles—can be tough, a sustainable way of continuing to live everyday life as a couple is reported as being facilitated by “working together” (Niedling & Hämel, 2023). In the present study, all participants had long relationships with persons with RA. This stability could indicate that supportive dyadic coping has contributed to the relationship and to a well-functioning everyday life.

Differences depending on type of relationship were noticed, as cohabiting partners expressed more frustration than individuals not sharing household. This is not surprising, since partners generally share activities in everyday life more closely than people not living together. In addition, living close to someone with inflammatory arthritis can create tension between providing assistance and respecting autonomy (Untas et al., 2020), a challenge evident in our participants' reflections on when to help and when to step back. Therefore, clear and tailored information is of importance, especially for partners. Longer relationships may provide more time to develop shared routines, knowledge and coping strategies, which could influence both informational needs and perceptions of involvement. However, relationship duration was not specifically explored in this study, and its influence cannot therefore be determined.

Experiences during different stages of diagnosis might differ. For example, Weitkamp et al. (2021) report that people with chronic physical illness have limited knowledge early in the disease process and, therefore, difficulties sharing experiences. This may influence how significant others experience involvement during the early stages of RA. Furthermore, family members of individuals with chronic illness are reported to undergo a five-stage process, starting with confusion at time of diagnosis, followed by inner conflicts regarding roles and activities, health and daily routine considerations, adjustment, and finally acceptance (Khalid et al., 2025). This may be linked to our participants' initial worries and misconceptions, followed by adaptations and perceived well-functioning everyday life several years after diagnosis.

In terms of significant others' perceptions of lacking involvement, policies and legislations, such as the Swedish Patient Act (Ministry of Health & Social Affairs, 2014) clearly state that significant others should be involved in the care of patients, in this case persons with RA, as long as it does not interfere with patient confidentiality, and as long as the patient does not object. However, based on the results of our study, it seems that this law is not always followed. On a national level, and as stated earlier, guidelines also expose shortcomings when it comes to involving significant others in treatment and rehabilitation of persons with RA (National Board of Health & Welfare, 2021). Our study highlights that more attention should be paid to involving and inviting significant others into the RA care and rehabilitation process, as suggested by previous research (Bergström et al., 2023). On an international level, research and guidelines regarding significant others have been encouraged by, for example, EULAR when it comes to patient education (Zangi et al., 2015). As RA is a chronic disease, and as the involvement of significant others is encouraged by both themselves as well as legislation, it is important to make them a part of care beginning at diagnosis, and, furthermore, to keep them continuously involved throughout the disease process. If the appropriate conditions are provided, significant others can contribute to the biopsychosocial approach (Primdahl et al., 2026), potentially reducing the strain on the healthcare system.

### Strengths and limitations

A major strength of this study is the involvement of research partners from early in the process. Their experiences have helped to ensure the importance and relevance of the results to significant others, as well as enrich the interpretation of themes during data analysis. Although patients' views would also have been of interest, no patients were invited as research partners, as focus of this study is to highlight the views of significant others specifically. We have aimed to give significant others a platform to express their own voices and experiences.

Another of this study's strengths is that persons with RA all come from an established and well-controlled cohort, TIRA-2. However, persons with RA have acted as gatekeepers in this case, since they have chosen the significant others, also meaning that some significant others have not been invited. This might indicate that the participants who agreed to take part in the study have good and grounded relationships, and it would be of interest to hear the voices of the less successful relationships, even though we can of course only speculate on the reasons for not inviting a significant other. Still, every significant other who was invited agreed to participate in the study.

One aspect that might have influenced the study's results is the clinical characteristics of the patients, and how these can impact the experiences of significant others. For example, Carpenter et al. (2015) reported that partners of patients with arthritis were more information-seeking as medication regimen was more complex, indicating more severe disease. The aspect of clinical characteristics was, however, not specifically addressed in this study, which can be considered a limitation.

Our sample consisted of different types of relationships, as well as both men and women, and included relationships of different lengths, which is considered a strength and something that enriched and nuanced the results. Although, the majority of our participants were female. As experiences differed slightly by gender, the results might have highlighted different aspects had more male participants been included.

We have discussed coping in relation to our results, which might take different shapes in different cultural settings. For example, Kayser et al. (2014) report cultural differences regarding involvement of extended family in illness experiences, as well as flexibility in relation to gender roles. In question of transferability of our results, we believe that our broad definition of “significant other” makes our findings relevant in other cultural settings, regardless of family structures.

### Implications for future research

It might be difficult for significant others to express what kind of support they want when information is lacking. It is therefore important to make significant others aware of the support available from the start, and particularly if the person with RA becomes increasingly ill. Consequently, it would be of interest to reach a larger sample of significant others, such as through a survey, in order to gain further information about how to better provide the information and support they need. Furthermore, as support needs may vary across the disease course, it would be of importance to specifically target significant others of newly diagnosed patients. In addition, since the persons with RA in our study all had access to modern treatment beginning at diagnosis, it would be of interest to look at differences between modern and earlier treatment, from the significant others' point of view. This could be achieved through longitudinal studies tracking adaptations and support needs over time. Additionally, it would be valuable to compare the experiences of significant others depending on patient clinical characteristics, such as low versus high disease activity.

## Conclusions

Significant others of persons with RA report lacking information and limited involvement in care and rehabilitation. Moreover, they express a need to make everyday life work in regard to the diagnosis, and stress that support is needed. This motivates increased focus on the significant others of persons with RA and their needs. Since RA is a chronic diagnosis, it is important to keep a longitudinal view on persons with RA as well as their significant others, as their well-being is of utmost importance in the lives—and the rehabilitation process—of persons with RA.

## Data Availability

The datasets generated and analyzed during this study are not publicly available due to the nature of ethical approval. Anonymized data are available from the corresponding author on reasonable request.
